# *UGT74S1* is the key player in controlling secoisolariciresinol diglucoside (SDG) formation in flax

**DOI:** 10.1186/s12870-017-0982-x

**Published:** 2017-02-02

**Authors:** Bourlaye Fofana, Kaushik Ghose, Jason McCallum, Frank M. You, Sylvie Cloutier

**Affiliations:** 1 Charlottetown Research and Development Centre, Agriculture and Agri-Food Canada, 440 University Avenue, Charlottetown, Prince Edward Island C1A 4N6 Canada; 2 Morden Research and Development Centre, Agriculture and Agri-Food Canada, 101 Route 100 Unit 100, Morden, Manitoba R6M 1Y5 Canada; 3 Ottawa Research and Development Centre, Agriculture and Agri-Food Canada, 960 Carling Avenue, Ottawa, Ontario K1A 0C6 Canada; 4 Department of Plant and Soil Science, Texas Tech University, Lubbock, TX 79409 USA

**Keywords:** Flax, Secoisolariciresinol, SDG lignan, Genome mining, Gene duplication, UGTs, Neofunctionalization

## Abstract

**Background:**

Flax lignan, commonly known as secoisolariciresinol (SECO) diglucoside (SDG), has recently been reported with health-promoting activities, including its positive impact in metabolic diseases. However, not much was reported on the biosynthesis of SDG and its monoglucoside (SMG) until lately. Flax UGT74S1 was recently reported to sequentially glucosylate SECO into SMG and SDG in vitro. However, whether this gene is the only UGT achieving SECO glucosylation in flax was not known.

**Results:**

Flax genome-wide mining for UGTs was performed. Phylogenetic and gene duplication analyses, heterologous gene expression and enzyme assays were conducted to identify family members closely related to *UGT74S1* and to establish their roles in SECO glucosylation. A total of 299 different UGTs were identified, of which 241 (81%) were duplicated. Flax UGTs diverged 2.4–153.6 MYA and 71% were found to be under purifying selection pressure. *UGT74S1*, a single copy gene located on chromosome 7, displayed no evidence of duplication and was deemed to be under positive selection pressure. The phylogenetic analysis identified four main clusters where cluster 4, which included *UGT74S1*, was the most diverse. The duplicated *UGT74S4* and *UGT74S3*, located on chromosomes 8 and 14, respectively, were the most closely related to *UGT74S1* and were differentially expressed in different tissues. Heterologous expression levels of UGT74S1, UGT74S4 and UGT74S3 proteins were similar but UGT74S4 and UGT74S3 glucosylation activity towards SECO was seven fold less than UGT74S1. In addition, they both failed to produce SDG, suggesting neofunctionalization following their divergence from *UGT74S1*.

**Conclusions:**

We showed that UGT74S1 is closely related to two duplicated genes, UGT74S4 and UGT74S3 which, unlike UGT74S1, failed to glucosylate SMG into SDG. The study suggests that UGT74S1 may be the key player in controlling SECO glucosylation into SDG in flax although its closely related genes may also contribute to a minor extent in supplying the SMG precursor to UGT74S1*.*

**Electronic supplementary material:**

The online version of this article (doi:10.1186/s12870-017-0982-x) contains supplementary material, which is available to authorized users.

## Background

Flax lignans are reported for a wide range of health benefits [1–3]. Various plant species have been shown to produce different types of lignans including secoisolariciresinol diglucoside (SDG) known as the main lignan in flax (*Linum usitatissimum* L.) seed [4, 5]. Lignans in flax seed are usually found in a glycosylated form, SDG, and ester-lined within oligomeric chains [6] making a macromolecule [7, 8]; its monomeric aglycone (SECO) and intermediate monoglucoside (SMG) forms not being accumulated in the seed. Glycosylation leads to the structural complexity and diversity of phytochemicals [9]. It ensures the stability and water solubility of plant natural products, reduces their reactivity or toxicity [10], and eases the transport and storage in the cells [11]. Glycosylation is achieved by CAZymes which include the glycosyltransferases (GTs) superfamily. GTs have been classified into 94 families, family 1 being referred to as uridine glycosyltransferases (UGTs) [12, 13]. In plants, UGTs have a 44 amino acid characteristic signature box commonly known as plant secondary product glycosyltransferase (PSPG) box [13, 14] and which role in ligand binding and catalysis has been documented in plants [10]. UGTs transfer UDP-activated sugars such as UDP-glucose to acceptor subtracts to form glucosides [15, 16]. In *Arabidopsis*, more than 120 UGTs have been reported and grouped into 30 sub-families based on sequence homology [13].

In flax, Barvkar et al. [17] recently reported 137 UGTs from the flax draft genome [18] and organized them into 14 groups (named A to N). However, no functional characterization was provided. Concurrently to Barvkar’s flax genome data mining study [17], we cloned and characterized five family 1 UGT genes (JX011632, JX011633, JX011634, JX011635, JX011636) and demonstrated that UGT74S1 (JX011632) was the only one able to glucosylate SECO by sequentially forming SMG and SDG [19]. Further, by performing 3D modeling of the UGT74S1 protein followed by ligand docking, targeted site-directed mutagenesis, heterologous expression and enzyme assays, Gln^337^ and Ser^357^ were found to be essential for the glucosylation of SMG into SDG, whereas Trp^355^ and His^352^ appeared to be critical for UGT74S1 glucosylation activity toward SECO in vitro [20]*.* However, whether UGT74S1 was the only UGT achieving the SECO glucosylation into SDG in the flax genome remained unknown.

Gene duplication is a common feature of many plant genomes. Evidence for ancient whole genome duplication events have been reported for flax [18], a finding supported by in-depth studies of duplication of fatty acid biosynthetic genes [21–23]. Similar studies on the UGT family have not yet been conducted in flax, despite the reported UGT diversity [17]. More specifically, the fate of *UGT74S1* through the flax genome evolution and its relation to other UGTs is unknown, as are the functional roles of its close relatives. The objectives of this study were to further study the diversity and evolution of UGT genes in flax, identify UGTs closely related to *UGT74S1,* and assess their functionality through glucosylation activity assays towards SECO for SDG formation.

## Methods

### Plant materials

Flax (*Linum usitatissimum* L.) seeds of cv AC McDuff [24] were planted in growing seasons 2008–2011 at AAFC Harrington farm (Harrington, PEI, Canada) and tissues were sampled as previously described [19].

### RNA and DNA isolation

Total RNA was extracted from developing flax seed, leaf and stem tissues using the Trizol RNA kit (Thermo Fisher Scientific, Burlington, ON, Canada) as previously described [25]. The extracted RNA samples were further purified using the PureLink™ RNA Mini kit (Thermo Fisher Scientific), quantified using the Qubit RNA HS Assay Kit (Thermo Fisher Scientific), and the quality was checked by agarose gel electrophoresis and Experion RNA analyzer (BioRad, Missisauga, ON, Canada) as previously described with slight modifications [19]. Genomic DNA was extracted from flax leaves using the Qiagen DNeasy Plant Mini Kit (Qiagen, Mississauga, Ontario), quantified using the Qubit dsDNA BR Assay Kit (Thermo Fisher Scientific), and the quality was verified by agarose gel electrophoresis.

### Genome-wide mining and phylogenetic analysis of UGTs

*UGT74S1* (JX011632), *UGT74T1* (JX011633), *UGT89B3* (JX011634), *UGT94H1* (JX011635), *UGT712B1* (JX011636) [19] and 137 previously identified flax UGTs [17] were used as queries to identify putative UGTs from the 43,471 annotated genes of the flax genome assembly [18] using BLAST with an E-value of 1e-10. The identified UGT candidates were further annotated by analyzing their gene structure and sequence similarity.

Phylogenetic analysis of genome-wide UGTs was performed using MEGA 6.0 [26]. The protein sequences for 68 out of 299 UGTs were found to be very short compared to others, probably truncated genes, and were excluded from the tree construction. To get a general overview of the tree topology from as many UGTs as possible, protein sequences of the remaining 231 UGT sequences were aligned in a first step using ClustalW [27] and a phylogenetic tree was constructed using the Neighbor-Joining (NJ) algorithm with 500 bootstrap replicates.

Because UGTs are characterized by a 44 amino acid PSPG motif signature box [13, 14] known to be involved in substrate recognition and catalysis [10, 11], UGT candidate gene list was narrowed down to only those carrying a PSPG motif. The 44 conserved amino acids of the PSPG motif and their 50 bp upstream and downstream sequences from 192 UGTs were extracted. These amino acid sequences were aligned with MUSCLE [28] and a phylogenetic tree was constructed using the maximum parsimony analysis method with 500 bootstrap replicates.

### Gene duplication analysis and UGT divergence

Gene duplication analysis was conducted by self-BLAST of the entire putative UGT database using a threshold E-value of 1e-30 [23]. Pairs of UGT genes returning reciprocal top hits of each other and having identical or highly similar gene structure were declared duplicate copies. The identified UGT genes were assigned to the 15 flax linkage groups, each representing a chromosome, based on the sorted flax genome sequence [29].

Duplication and divergence times were determined from the ratio of calculated non-synonymous substitution (*Ka*) over the calculated synonymous substitution (*Ks*) values [29]. Briefly, 192 full-length cDNA sequences (excluding all partial sequences) were aligned in Mega v7.0 [26] and the *Ka/Ks* ratio was calculated [30]. *Ks* values lower than 0.001 were removed from the dataset as suggested by Sveinson et al. [31]. Furthermore, *Ks* values larger than 2 were excluded to minimize the saturation effects [32]. The evolutionary distance between pairs of genes was determined based on the *Ks* corrected with the Nei-Gojobori model of nucleotide evolution which accounts for multiple substitutions per site 26]. The divergence (*k*) of a pair of duplicated genes was converted into duplication or divergence time (*t*) in million years (MY) following the equation *t* = *k*/(2*r*)/10^6^, where *r* is the substitution rate of 6.5 × 10^−9^ substitutions per synonymous site per year [33].

### Functional analysis of *UGT74S1* and its closely related UGTs *Lus10006353* and *Lus10014148*

To assess the functionality of the two UGTs most closely related to *UGT74S1*, gene splicing of genomic DNA was performed to recover the full length coding sequence (CDS) of *Lus10006353* and *Lus10014148* which have short single introns [17, 18]. Tissue-specific and heterologous gene expression studies of these two genes were undertaken alongside *UGT74S1*.

### *Lus10006353* and *Lus10014148* gene splicing from genomic DNA

Extension of overlapping gene segments by PCR is a simple technique for gene splicing (Additional file 1) ([34], http://www.methods.info/Methods/Mutagenesis/PCR_splicing.html). To delete the intron from the genomic DNA through joining the two exons together, genomic sequences corresponding to *Lus10006353* (JN088326.1) and *Lus10014148* (JN088327.1) were used to design gene specific primers (Additional files 1 and 2). For each sequence, an external flanking primer pair was designed from the 5′ and 3′ end of the CDS, referred to as exonic forward and reverse primers. Additionally, two 30-nucleotide internal primers were designed. The first internal primer called exon I-R consisted of the reverse complement to the first 15 nucleotides in the sense strand of exon II (+15 bp downstream of the intron) and the last 15 nucleotides of the reverse strand of the exon I (−15 bp downstream of the intron). The second internal primer called exon II-F consisted of the last 15 nucleotides in the sense strand of exon I (−15 bp downstream of the intron) and the first 15 nucleotides of exon II sense strand (+15 bp downstream of the intron) as previously described (http://www.methods.info/Methods/Mutagenesis/PCR_splicing.html) (Additional file 1).

The gene splicing was conducted in three steps. The initial step was the amplification of the two exons using the 5′ exonic I forward and exon I-R in a first reaction, and exon II-F and 3′ exonic II reverse in a second reaction. DNA was diluted and 3 μL aliquots (10 ng/μL) were used as template in subsequent PCR reactions. PCR cycles consisted of an initial denaturation at 94 °C for 2 min followed by 30 cycles of 94 °C for 30 s, 55 °C for 45 s and 72 °C for 90 s prior to a final extension at 72 °C for 10 min. Aliquots of 6 μL of the PCR products were resolved on 1% agarose gels stained with ethidium bromide. The amplified exon fragments I and II were purified using the QIAquick PCR purification kit (Qiagen), diluted 50 folds and 4 μL aliquots of each purified product were pooled and used as template in the second gene splicing step where full length templates for each gene were generated. During PCR, overlapping strands of the two intermediate products form a duplex, providing *Taq* with a free 3′end for extension and a single strand for polymerization, thereby generating a single full-length product. This PCR reaction consisted of 10 cycles at 95 °C for 30 s, 25 °C for 45 s, and 72 °C for 90 s. The resulting PCR product was diluted 50 folds and 3 μL aliquots were used as a template in a third and final PCR reaction conducted at 94 °C for 3 min followed by 30 cycles of 94 °C for 30 s, 55 °C for 45 s and 72 °C for 90 s, followed by a final extension at 72 °C for 10 min. The fused full length fragments were purified with the QIAquick gel extraction kit (Qiagen), cloned into TOPO TA vector and transformed in *E. coli* (Thermo Fisher Scientific) similar to previous descriptions [19].

### *UGT74S1, Lus10006353* and *Lus10014148* gene expression in different flax tissue

To quantify the gene expression levels of *Lus10006353* and *Lus10014148* in different flax tissues (developing flax seed, root, leaf, and stem), real-time PCR primers were designed from the 3′ end of *LuS10014148, LuS10006353, UGT74S1,* and a ribosomal RNA (EU307117), the latter being used for data normalization (Additional file 2) as performed in a previous report [19]. First strand cDNA synthesis and real-time PCR conditions were as previously described [19]. The output gene expression data were generated using the 2^-∆∆CT^ method [35], and the results were presented as fold changes expression relative to that of 0 day after anthesis (DAA) for all tissues [19].

### Cloning and heterologous expression

The TOPO TA cloned full length fragments for *Lus10006353* and *Lus10014148* were sequenced for confirmation. Flanking primers carrying restriction enzyme sites (Additional file 2) were designed to shuttle the full length *Lus10006353* and *Lus10014148* into the yeast (*Saccharomyces cerevisiae*) expression vectors pYES2/NT C and B (Thermo Fisher Scientific), respectively. The pYES2/NT C construct carrying the full-length cDNA for *UGT74S1* was previously described [19]. All three constructs were transformed in the yeast (*Saccharomyces cerevisiae*) strain INVSc1 following manufacturer’s instructions (Thermo Fisher Scientific) and single transformant colonies were cultured, induced, harvested and lysed as previously reported [19, 20]. Protein expression for each of the three UGTs, including UGT74S1 as control, was monitored by western blot using equal amount of proteins and diluted antibodies raised against the Xpress^TM^ epitope present between the 6× histidine tag and the multiple cloning site as previously described [19, 20].

### Enzyme assays and reaction products determination and quantitation

To determine whether the two UGTs (LuS10014148 or LuS10006353) could glucosylate SECO into SDG as reported before for UGT74S1 [19, 20], the purified native proteins obtained from the yeast cultures expressing UGT74S1, LuS10014148, or LuS10006353 were reacted with SECO (Chromadex, Irvine, CA, USA), the only substrate relevant to this study, in the presence of UDP-glucose [19, 20] or UDP-galactose. The 100 μL reaction mixture composition and reaction incubation conditions were exactly as described in our previous report [20].

For separation, identification, and quantitation of the reactants and their products, a Waters H-Class Acquity UPLC system (Waters, Milford, MA, USA) equipped with a TQD tandem mass spectrometer (Waters) and a Waters CSH C18 column (100 mm × 2.1 mm, 1.8 μm particle size) were used as previously described [19, 20], albeit with slight modifications. In addition to MS2 scanning mode, selected ion recording (SIR) spectra were collected to improve the detection sensitivity of SECO, SMG, SDG and SECO monogalactoside (SMGal). The capillary voltage was set at 3 kV, the extractor at 3 V, and RF lens at 0.1 V. The chromatographic parameters followed a binary gradient system composed of 3% formic acid in water (A) and acetonitrile (B), varying according to the following program: t0, A = 98%; t1 = 4.4 min, A = 0%; t2 = 6 min, A = 0% isocratic; t3 = 7 min, A = 98%; t4 = 8 min, A = 98% isocratic. Peaks detected at 280 nm, indicative of phenolic compounds, and were validated using authentic standards (SECO and SDG) purchased from Chromadex (Chromadex, Irvine, CA, USA) as described in [19]. A standard curve for SDG was created using the SDG standard described above. Purified SMG standard was prepared in-house as previously described [19, 36].

All reactions were carried out in triplicates and the data are presented as the means ± standard deviations. A one-tailed student’s *t*-test was performed to test the statistical significance of metabolite production levels by UGT74S1, Lus10014148 and Lus10006353 [20].

## Results

### UGT gene duplication and diversity in the flax genome

Genome-wide mining for UGT genes identified 299 non-redundant sequences dispersed on all 15 flax chromosomes (Additional files 3 and 4). With a mere ten sequences, chromosomes 12 had the fewest UGT genes whereas chromosome 1 harbored the most with 35 (Additional files 3 and 5). A total of 241 (81%) of the identified UGT genes were duplicated, of which 56 (23%) corresponded to intra-chromosomal duplications (Additional file 3). Fourteen (25%) of those occurred on chromosome 1 whereas no intra-chromosomal duplications were observed on chromosomes 2, 7, 10, 11 and 14 (Additional file 3). *UGT74S1* was located on chromosome 7 and displayed no evidence of gene duplication.

Phylogenetic analysis from the 231 out of the 299 UGTs, excluding 68 truncated sequences, revealed four main clusters (Additional file 6) and 15 sub-clusters. Cluster 4 was the most diverse, accounted for 53% (122/231 UGTs) of the UGT genes, and included eight (F, G, H, I, J, K, L, and N) of the 14 groups previously reported [17]. The fifteenth new group identified in this study was hereafter named “O” (Additional file 6). *UGT74S1* clustered within group L as previously reported and belonged to a small gene family of five UGTs including *Lus10017825* (JN88324/JX011632), *Lus10006353* (JN088326)*, Lus10014148* (JN088327)*, Lus10024486,* and *Lus10008742* (JN088325), supported by a 53% bootstrap value and hereafter called the Lus10017825 subclade. Two members of this subclade, *Lus10014148* and *Lus10006353,* were shown to be duplicated genes located on chromosomes 8 and 14, respectively, and were the most closely related to *UGT74S1* (Additional files 3 and 6). This small gene family clustered with a sister subclade, herein called Lus10006352/Lus10006351 subclade, that was supported with a 51% bootstrap value and which included *Lus10006352* (JN088329)*, Lus10006721* (JN088328), *Lus10006351* and *Lus10006720* (Additional file 6).

**Fig. 1:**
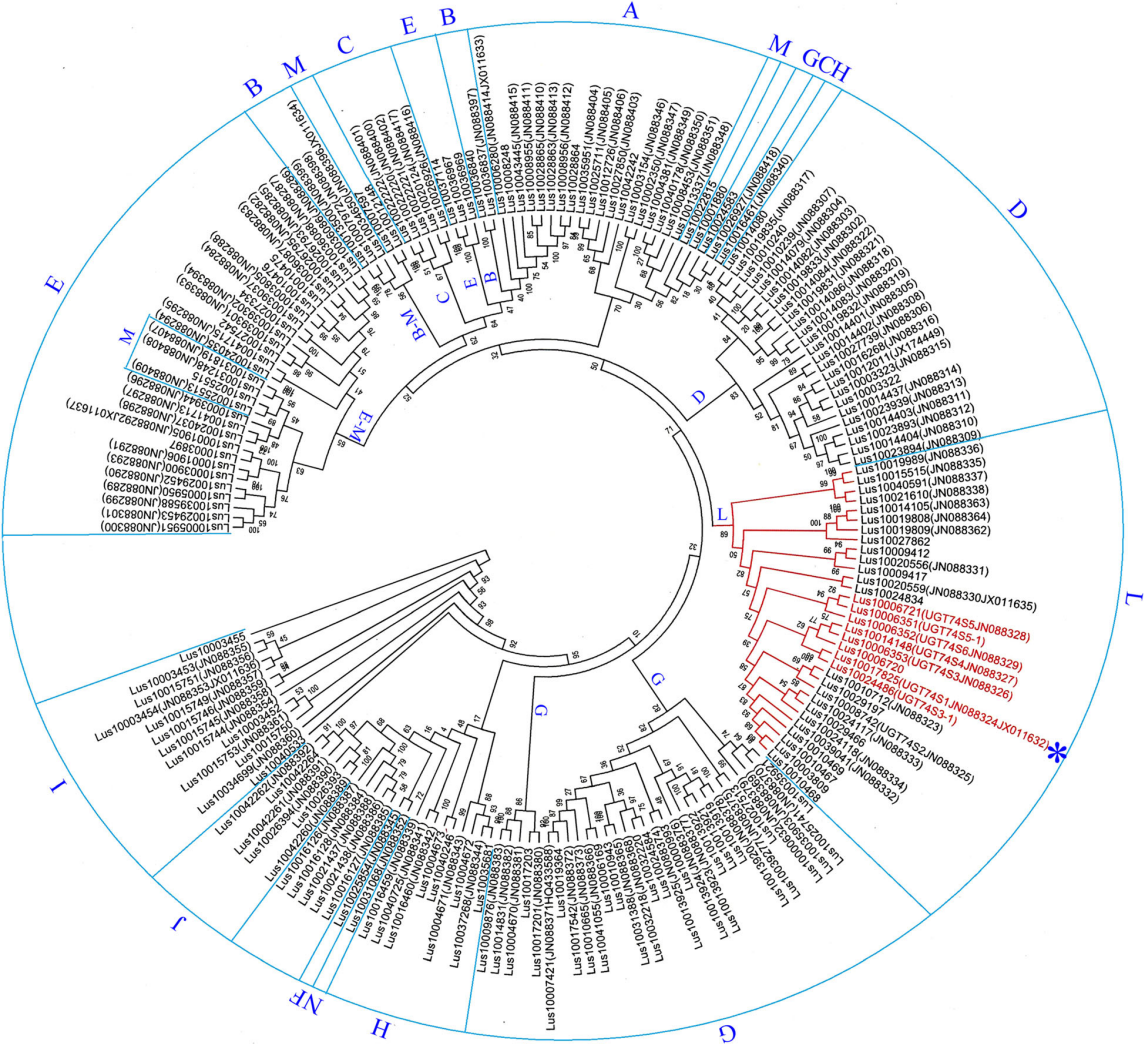
Phylogenetic relationship among 192 flax UGTs based on the conserved PSPG motif and its surrounding regions. The different groups are indicated by alphabetic letters *A*-*O* around and inside the circular tree. *UGT74S1* belongs to a small gene family highlighted in *red* within group L and is marked with an *asterisk*

To further assess the consistency of the observed groupings, a new tree was constructed using a conserved region from 192 UGT sequences. Consistent with the first large phylogenetic tree, this reduced tree showed, with relatively high bootstrap values (10–100%), the same configuration and groupings as previously reported [17]. However, the A, B, C, E and M groups were not as well resolved as in the previous tree. In this second phylogenetic tree, group “L” was well supported by an 89% bootstrap value and the Lus10017825 subclade comprised *Lus10017825* (JN88324/JX011632), *Lus10006353* (JN088326)*, Lus10014148* (JN088327)*, Lus10024486* and *Lus10006720* while the Lus10006352/Lus10006351 subclade included *Lus10006352* (JN088329)*, Lus10006721* (JN088328) and *Lus10006351* (Fig. 1), thus providing more confidence in our phylogeny for inferring the evolutionary history of the UGTs. Following UDP-glucosyltransferase nomenclature, *Lus10006353, Lus10014148, Lus10024486, Lus10008742*, *Lus100Lus10003520352, Lus10006721* and *Lus10006351* were annotated as members of *UGT74S3*, *UGT74S4*, *UGT74S3-1*, *UGT74S2*, *UGT74S6*, *UGT74S5*, *UGT74S5-1* sub-families, respectively. Therefore, *Lus10014148* and *Lus10006353* will be hereafter designated by their UGT nomenclature: *UGT74S3* and *UGT74S4,* alongside with *UGT74S1* (*Lus10017825*).

### UGT gene divergence

**Fig. 2:**
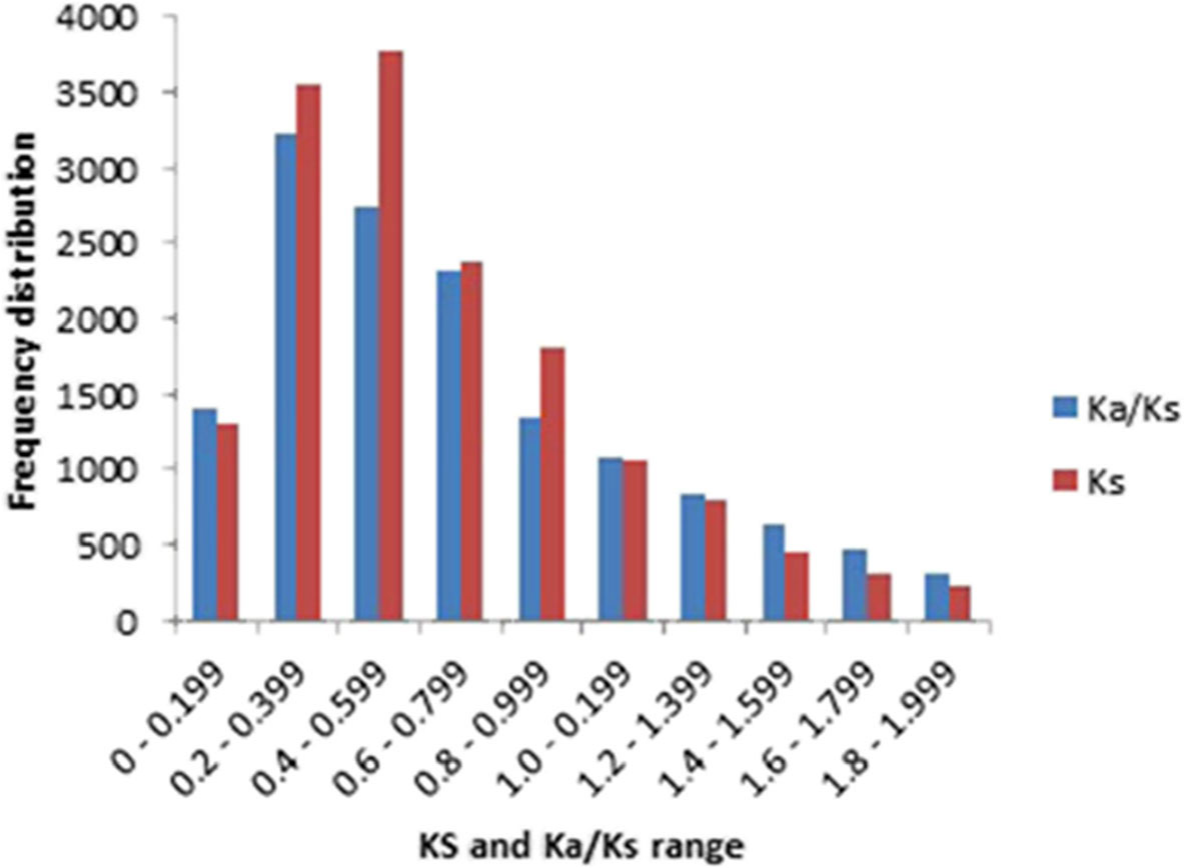
Frequency distribution of Ks values and *Ka/Ks* ratios among 15,616 UGT gene pairs obtained from 192 UGT sequences

We examined UGT sequence divergence by determining the *Ka/Ks* ratio among 192 UGT sequences carrying the PSPG motif. After *Ks* calculation and data filtration, 15,616 (85%) UGT gene pairs with *Ks* ranging between 0.031 and 1.997 were retained. Overall, 82% of the gene pairs had *Ks* values <1, including 45% with *Ks* values <0.5. The *Ks* mean value was 0.642 and the distribution peaked at 0.493 (Fig. 2). Similarly, 47 and 71% of the UGT gene pairs had *Ka/Ks* < 0.6 and *Ka/Ks* < 1, respectively. Less than 1% of the gene pairs had *Ka/Ks* = 1 (Additional file 7). The distribution frequency of the individual *Ka/Ks* values peaked at 0.306 (Fig. 2).

The gene pairs involving the SDG lignan-forming gene *UGT74S1* (*Lus10017825*) had *Ks* < 1 and *Ka/Ks* < 1 in a proportion of 85 and 56%, respectively. Within the Lus10017825 and Lus10006352/Lus10006353 subclades, all *Ks* derived from gene pairs involving *UGT74S1* ranged from 0.208 to 0.264. In contrast, only two of the eight gene pairs (25%) had *Ka/Ks* < 1, the remaining 75% of the gene pairs displayed *Ka/ks* >1.

The divergence time between pairs of the 192 UGT genes ranged from 2.4 to 153.6 MYA (Additional file 7). The divergence time between the 31 genes clustered in group L spread from 7.4 to 148 MYA (Additional file 8). Within the Lus10017825 and Lus10006352/Lus10006351 subclades, *Lus10024486* (*UGT74S3-1*) and *Lus10006352* (*UGT74S2)* appeared to be the most ancient, diverging from one another approximately 42 MYA. *UGT74S1* (*Lus10018725*) may have diverged from the other seven UGTs clustered in these two subclades around 16–20 MYA. Based on our inferences, *UGT74S3* (*Lus10006353)* probably duplicated from *UGT74S4* (*Lus10014148)* ~15 MYA. The evolutionary relationships between *UGT74S5* (*Lus10006721), Lus10006720, UGT74S6 (Lus10006352)* and *UGT74S5-1 (Lus10006351)* were found to be complex. The duplication study identified both *UGT74S6* (chr 8) and *UGT74S5-1* (Chr 8) as duplicates of *UGT74S5* (Chr 5) on one hand, and also *UGT74S6* (chr 8) as a duplicate of *Lus10006720* (Chr 5) on the other hand, with duplication/divergence times of about 16 and 15.5 MYA for *UGT74S5/UGT74S5-1* and *Lus10006720/UGT74S6*, respectively. However, the divergence time for the *UGT74S5/UGT74S6* pair could not be determined using the filtration criteria set (Table 1).

**Table 1:** Pairwise matrix of flax UGT divergence time in MYA for the 8 UGT genes clustered Lus10017825 and Lus10006351/Lus1006352 subclades within the group “L” of the phylogenetic tree. The most recent and ancient divergence times are highlighted in blue and green, respectively

	Lus10006721	Lus10006351	Lus10006352	Lus10014148*	Lus10006353*	Lus10006720	Lus10017825*
Lus10006351	16.01						
Lus10006352	N/A	18.25					
Lus10014148	33.28	35.95	38.26				
Lus10006353	15.74	16.90	17.89	14.97			
Lus10006720	13.84	14.73	15.47	13.24	N/A		
Lus10017825*	17.55	19.00	20.27	16.59	N/A	16.01	
Lus10024486	35.95	39.10	41.88	16.01	16.01	14.05	17.89

N/A, denotes that the gene pairs did not meet the filtration criteria set in Methods

*UGT74S1* (*Lus10017825*), *UGT74S3* (*Lus10006353*), and *UGT74S4 (Lus10014148*) are indicated by an asterisk

### Structural gene organization of *UGT74S1* and its closest relatives

*UGT74S1* is a 1407 bp gene with a single intron of 739 bp [19]. To assess the functionality of the two UGTs that were the most closely related to *UGT74S1*, i.e., *UGT74S3* and *UGT74S4*, we obtained their full coding regions by gene splicing. *UGT74S3* generated a spliced fused CDS of 1416 bp by joining the exons of 678 and 738 bp from a 1492 bp genomic DNA fragment. Similarly, a spliced fused CDS of 1371 bp was obtained by joining the exons of 643 and 728 bp from a 1464 bp genomic DNA product for *UGT74S4* (Additional file 9). The amplified products were of expected sizes as estimated from the original sequences in the NCBI database (JN088326.1; JN088327.1) and in Phytozome (Lus10006353.g, Lus10014148.g). In this study, *UGT74S3* and *UGT74S4* had 76 and 93 bp introns, respectively and their fused spliced coding sequences were confirmed as 100% identical to those already reported (Additional file 10). It is noteworthy to mention that *UGT74S4*, corresponding to NCBI accession JN088327.1, was reported by Barvkar et al. [17] with a 27 bp truncation at its 5′ end (Additional file 10). This truncation was detected by comparing the amplified genomic and spliced fused sequences of *UGT74S4* with that of JN088327.1 [17] and of Lus10014148.g from the flax draft genome (http://phytozome.jgi.doe.gov/pz/portal.html#!gene?search=1&detail=1&method=3127&searchText=transcriptid:23152916). In *UGT74S3*, the intron is located between nucleotide positions 679 and 756, whereas it is located between nucleotide position 643 and 737 of *UGT74S4* (Additional file 10). Thus, the full genomic sequence size for *UGT74S4* is 1464 bp and not 1437 bp as previously reported [17]. The spliced full length cDNAs for *UGT74S3* (1416 bp) and *UGT74S4* (1371 bp) were predicted to code for 471 and 456 amino acids, respectively, and as also observed in this study (Additional file 11). Both showed nearly 62% identities to *UGT74S1* and displayed 80% identity to one other at the amino acid levels. The conserved 44 amino acids PSPG motif (Additional file 11) was located between amino acids 331 and 374 in UGT74S3 and between 343 and 386 in UGT74S4. The PSPG motif of UGT74S3 and UGT74S4 shared 90 and 80% identity with UGT74S1, respectively (Additional file 11). Four amino acids found to be conserved within the PSPG motif of UGT74S3 and UGT74S4 were substituted in UGT74S1: E339Q, A345K, M362L and W374C. UGT74S3 had three unique amino acids changes (L350V, R373E and T375G) and UGT74S4 had two (Q373E and N375G; Additional file 11).

### *UGT74S1*, *UGT74S3* and *UGT74S4* gene expression in different flax tissues

**Fig. 3:**
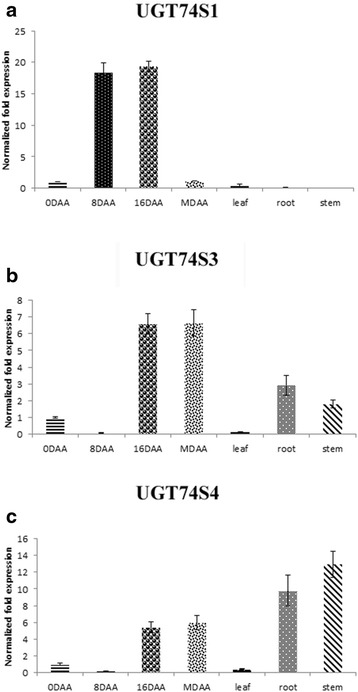
Expression profile for *UGT74S1*, *UGT74S3* (Lus10006353) and *UGT74S4* (Lus10014148) in developing seeds sampled at 0, 8 and 16 days after anthesis (DAA) and at maturity (MDAA), as well as leaf, root and stem tissues of flax cultivar AC McDuff. **a**, *UGT74S1*; **b**, *UGT74S3* (Lus10006353); and **c**, *UGT74S4* (Lus10014148)

Real time gene expression levels of *UGT74S4* and *UGT74S3* were similar in developing seeds and leaves but were markedly contrasting in stems and roots. Moreover, their expression patterns differed from that of *UGT74S1* in all tissues (Fig. 3). In developing seeds, *UGT74S1* expression mimic a bell curve pattern, peaking at 16 DAA (Fig. 3a) as previously observed [19]. At eight and 16 DAA, *UGT74S4* and *UGT74S3* expression levels were 3–260 times lower than *UGT74S1* whereas their expression levels were higher (5–6 times) than that of *UGT74S1* at maturity (Fig. 3b-c). In contrast, *UGT74S1* was barely expressed in root and stem tissues where *UGT74S4* and *UGT74S3* were highly expressed. Expression of all three UGT genes was low in leaves (Fig. 3).

### Heterologous protein expression and enzyme activity

**Fig. 4:**
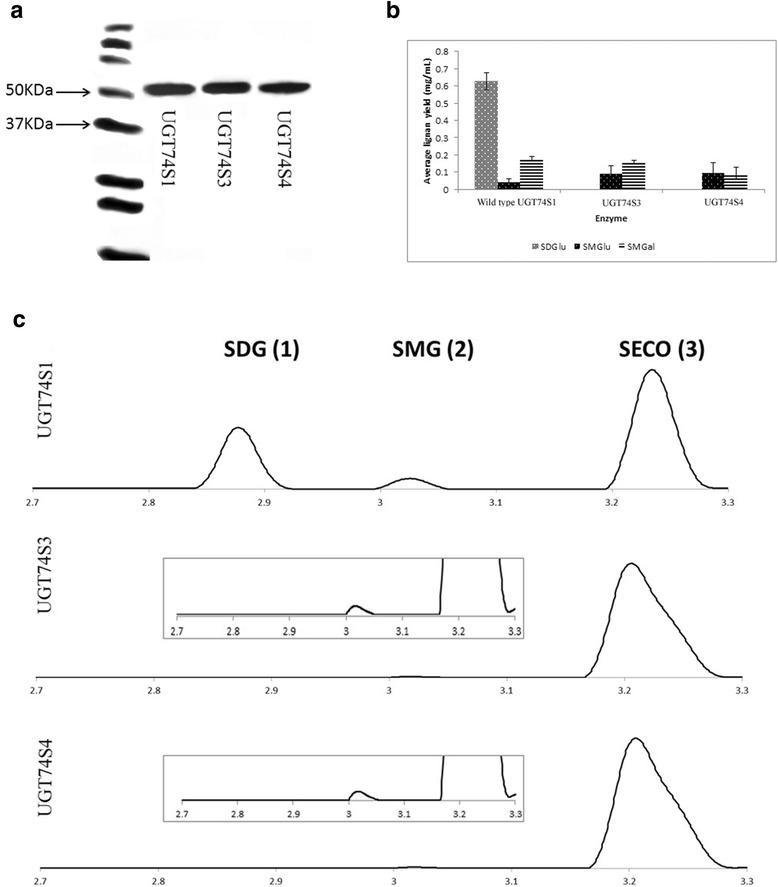
Western blot of UGT74S1, UGT7 4S3 (Lus10006353) and UGT74S4 (Lus10014148) and reaction products observed from enzyme assays using purified native enzymes. **a**, Western blot of the HisTag-purified UGT74S1, UGT74S3 and UGT74S4 probed using AntiXpress^TM^ antibody. The 37 and 50 kDa protein molecular marker sizes corresponding to the Western C precision plus protein marker mixed with conjugant (BioRad) are pinpointed. **b**, Reaction products formed by each of UGT74S1, UGT74S3 and UGT74S4 after enzyme reaction with SECO as substrate and UDP-glucose or UDP-galactose as sugar donor. *SDG* secoisolarciresinol diglucoside, *SMG* secoisolariciresinol monoglucoside, *SMGal* secoisolariciresinol monogalactoside. **c.** UPLC chromatograms (280 nm) showing the reaction products of 50 μg of purified proteins for the three UGTs using SECO as substrate and UDP-glucose as sugar donor. *Insets* show 10× magnification to visualize trace peaks of SMG in UGT74S3 and UGT74S4 reaction products. Appreciable accumulation of SDG (peak 1) and SMG (peak 2) was only observed for UGT74S1. The unreacted SECO (peak 3) is present in all three chromatograms

In this study, we wanted to assess whether the *UGT74S4* and *UGT74S3* enzymes had the ability to glycosylate SECO as reported for UGT74S1 [19, 20]. Thus, protein expression and glycosylation activity toward SECO were assessed for UGT74S4 and UGT74S3 in comparison with UGT74S1. The full-length cDNAs for *UGT74S4* and *UGT74S3* were expressed in yeast as previously described for *UGT74S1*. The western blot analysis showed that all three proteins highly expressed after 8 h of induction with 2% galactose (Fig. 4a). UGT74S4 and UGT74S3 showed strong intensity signals comparable to the 56.4 kDa of UGT74S1 previously reported [19, 20] and produced 54.5 and 56.1 kDa proteins, respectively, in agreement with their predicted sequences (Fig. 4a).

To determine the glucosylation activity of UGT74S4 and UGT74S3, enzyme assays were performed using the purified proteins, with UGT74S1 as a positive control (Fig. 4b-c). Although UGT74S4 and UGT74S3 protein expression was similar to UGT74S1 (Fig. 4a), their glucosylation activity towards SECO was seven fold less and both failed to produce SDG under our experimental conditions (Fig. 4b-c). No significant difference (*P* = 0.46) in glycosylation activity was observed between UGT74S4 and UGT74S3 towards SECO.

By using UDP-galactose as an alternative sugar donor for the production of SECO glycosides, all three proteins formed trace amount of SECO monogalactosides (SMGal). But no SECO digalactosides were detected with any of the three enzymes tested (Fig. 4b-c). No difference was observed among all three proteins with regards to the SMGal levels when UDP-galactose was used as alternate sugar donor.

## Discussion

UGTs are a large and complex family of enzymes that catalyze glycosidic bond formation [19]. Flax UGT74S1 has recently been reported as an enzyme sequentially glucosylating SECO into its monoglucoside SMG and diglucoside SDG when expressed in yeast [19, 20]. To get more insights into the exclusivity of UGT74S1 to achieve SECO glucosylation into SDG in flax, genome-wide mining, tissue-specific and heterologous gene expression, and enzyme activity assays were conducted. The study confirmed our previous findings [19, 20] and further shows that UGT74S1 may be the key enzyme controlling SECO glucosylation in flax, although UGT74S4 and UGT74S3 may also contribute to a minor extent in supplying SMG as substrate to UGT74S1 for the second glucosylation step*.*

A total of 299 UGTs were found dispersed throughout the ~373 Mb flax genome [18], corresponding to a gene density of 0.80 UGT/Mb, which is similar to *Arabidopsis* (0.79) but higher than rice (0.46), apple (0.32), grapevine (0.37), poplar (0.37), soybean (0.16) and sorghum (0.25). The percentage of UGTs among the total predicted genes in flax is 0.69%, higher than previously reported in dicots (0.32–0.54%) and falls in the same range as monocots (0.65–0.72%) [14].

Gene duplication is a common phenomenon in eukaryotes, such as plants [37, 38]. It leads to genetic novelty and diversity, evolution, and functional diversification [39–41], phenomena that are required by vascular plant species for adaptation and plasticity [13]. Paleoploidy events including whole genome duplications have been described in the genus *Linum* [31] and are considered key drivers of gene duplication [42–44]. Previous studies predicted 43,484 genes in the flax genome, of which 9920 genes were predicted to be duplicated [18]. Of the 299 UGTs identified in this study, 241 (81%) were found to be duplicated and accounted for 2.4% of all duplicated genes of the flax genome.

*Ks* has been previously used as a time indicator for duplication events, and its frequency distribution was reported to reflect the relative time of genome duplication [33, 45, 46]. Accordingly, the *Ks* calculated from the UGT gene pairs peaked at 0.493, and ranged from 0.4 to 0.6, indicating ancient duplications [46] during UGT evolution. Using the *Ks* values, the divergence among the pairs of 192 genes was estimated to have occurred 2.4–153.6 MYA. Likewise, the divergence time between the 31 genes clustered in the L group spans from 7.4 to 148 MYA. These results suggest that some flax UGT duplication events may have taken place prior to the most recent whole genome duplication estimated at 5–9 MYA [31]. Our *Ka/Ks* analyses showed that 71% of the UGT gene pairs had *Ka/Ks* < 1, indicating that most of the flax UGTs have evolved under a purifying (negative) selection [47]. About a quarter (28%) of the gene pairs had *Ka/Ks* > 1, thus being under positive selection pressure whereas 0.75% of the UGT genes found to be under neutral selection [46]. In our study, full protein sequences carrying PSPG motif were used to calculate *Ka* and *Ks.* Similarly, full length gene sequences have been used to determine the *Ks/Ka* values for the wheat and rice homologues [48]. Whether potential biases may be associated with the conserved domain’s own divergence rate, it is reasonable to assume that such bias, if any, should be evenly distributed and minimal because all the considered sequences had the PSPG motif and, thus, would not affect to the drawn conclusions.

Despite the high rate of UGT duplication in flax, *UGT74S1* gene appeared as a single copy gene located on linkage group 7 (chromosome 7). This gene was found to be involved in 160 gene pairs based on the criteria set in this study, of which 56% had *Ka/Ks* < 1 and 44% with *Ka/Ks* > 1, almost a 50/50 ratio for negative and positive selection. When a single member of a gene family is undergoing positive selection, it shows high *Ka/Ks* ratios when paired with all members of its family [49]. This fact was substantiated within the Lus10017825 and Lus10006352/Lus10006353 subclades where 75% of the gene pairs involving *UGT74S1* had *Ka/ks* >1, indicating that *UGT74S1* is under positive selection and, as such, it is experiencing reduced selective constraints [50] to remain a singleton [51]. Positive selection has been reported for *UGT73C10* and *UGT73C11*, playing a role in saponin-mediated insect resistance [52], and lignans, which have also been reported to take part in plant defense against insect [53, 54]. It is possible that *UGT74S1* was duplicated during one or both of the proposed ancient duplication events and, that its paralog(s) were subsequently deleted through gene loss as previously reported [47]. Single copy genes have been evoked to play key roles in maintaining essential metabolic processes in plants through macromolecular complex formation [51]. *UGT74S1* may have been maintained as a single copy gene in flax for SECO glucosylation into SDG lignans during adaptive divergence as was reported for *UDP-glucose pyrophosphorylase* in *Sorghum* [52].

Phylogenetic studies of UGTs have previously described 14 and 17 distinct groups using 107 UGT amino acid sequences from *Arabidopsis* [15] and 1520 UGTs from nine higher plants [14], respectively. In flax, Barvkar et al. [17] described 14 groups based on 137 UGTs. The 15 sub-clusters observed in the current study included the 14 groups previously reported in flax [17] and identified a new group named “O”. The grouping observed here is comparable to the 14–17 groups previously reported [14, 15, 17] and highlights the diversity of UGTs in flax, possibly through concerted evolution, a phenomenon also known in other higher plants [14].

Duplicated genes may face three different fates: nonfunctionalization in which one copy becomes silenced; neofunctionalization where one copy acquires a novel, beneficial function while the other copy retains the original function; or subfunctionalization where both copies become partially compromised by the accumulation of mutations [39]. *UGT74S4* and *UGT74S3*, which originated from a duplication event, were the closest to UGT74S1 that glucosylates SECO into SDG [19, 20]. Because high sequence similarity does not necessarily translate into similarity in expression and function, *UGT74S4* and *UGT74S3* tissue-specific expression and protein functionality towards SECO were investigated. Contrary *to UGT74S1*, *UGT74S4* and *UGT74S3* were barely expressed in the seeds at 8–16 DAA developmental stages, when SDG lignan synthesis increases rapidly [19, 55]. But, these 2 duplicated genes were more expressed in vegetative tissues and in mature seeds (a stage when SDG synthesis is already completed [56]) than *UGT74S1*, and showed different expression patterns, perhaps because of their tissue-specific promoters or their neofunctionalization after the duplication events [57]. The reported data suggest that these 2 genes may play major roles in other biological processes yet to be elucidated, as opposed to SECO glucosylation into SDG. High protein expression levels were achieved in the heterologous yeast expression system but under the conditions described in this study, UGT74S4 and UGT74S3 were unable to convert SECO into SDG. However, they were able to produce small amounts of SMG with an overall glucosylation activity towards SECO seven times lower than UGT74S1 suggesting that UGT74S4 and UGT74S3 proteins may prefer a different substrate. In contrast, UGT74S1 consistently produced higher amounts of SDG along with small amounts of SMG and, its gene was more highly expressed in developing seeds where SDG lignans accumulate as previously reported [19, 20]. These observations suggest that UGT74S1 may be the key functional player in SECO glucosylation into SDG, due to its ability to achieve both required glucosylation steps; first glucosylating SECO into SMG and then SMG into SDG ([19], and this study). Additionally, mutations in *UGT74S1* lead to reduced or abolished SDG production in vitro [20], further demonstrating its essential function. While not all the 299 UGTs reported in this study were biochemically evaluated for their ability to glucosylate SECO, the finding that *UGT74S4* and *UGT74S3* (the most closely related UGT genes to *UGT74S1)* produced SMG from SECO at a very low rates and failed to form any SDG, indicates that these two genes are not as essential as UGT74S1, which performs both required steps [19]. Although involvement of other cryptic UGTs in the SECO glucosylation process leading to SDG has not been exhaustively ruled out, one may question the evolutionary advantages of maintaining redundant genes and duplicated pathways for the same biochemical function. Moreover, recent evidence coming from a follow-up study conducted in our laboratory and focusing on TILLING of ethyl methane sulfonate (EMS) flax mutant lines support the idea that *UGT74S1* is unique in controlling SECO glucosylation into SDG (data not shown). In the current study, UGT74S4 and UGT74S3 were able to glycosylate SECO with UDP-glucose or UDP-galactose at low efficiency, forming SMG or SMGal respectively, under the described conditions, but failed to do so for SDG as did UGT74S1, and therefore, no further biochemical characterizations or optimizations were performed for these two enzymes. Indeed, if UGT74S4 and UGT74S3 were able to glycosylate SMG into SDG, even at a low rate, one would expect to observe trace SDG peak using the very sensitive MS detection methods, even under standard conditions, which would have warranted further biochemical optimization of the enzyme reactions. However, this was not the case under our experimental conditions, suggesting that these two proteins may not have the ability to perform the second glycosylation step. These enzyme activity data are in line with the conclusions drawn from the differential temporal gene expression data in the developing seeds, and reinforce the idea that UGT74S3 and UGT74S4 may play other roles in different metabolic processes apart from SDG lignan glucosylation. The role played by the PSPG motif in ligand binding and catalysis has been well-documented in plants [10, 58]. The variations within the PSPG observed between UGT74S1 and its two closely related UGT genes may be responsible for the differences in their substrate specificity as previously reported [20] and may account for the observed differential functionality.

## Conclusions

UGTs form a large gene family where the majority of the members are duplicated in the flax genome and subject to negative or purifying selection pressures whereas UGT74S1 is a singleton that has evolved under positive selection pressure. Flax *UGT74S4* (*Lus10014148*) and *UGT74S3* (*Lus10006353*) were the most closely related to *UGT74S1* but their encoded proteins were unable to glycosylate SMG into SDG. Whereas not all UGTs in the genome were biochemically tested in this study, it is unlikely that other redundant UGT genes having the same roles in SMG glucosylation as UGT74S1 would be maintained without a specific evolutionary advantage. However, the possibility of cryptic UGT functionality cannot be totally ruled out. Nonetheless, the current study provides additional insights into UGT gene duplication events in flax and could be of interest to plant evolutionary biologists, breeders and plant natural product chemists.

## Additional files

Additional file 1: Figure S1. Schematic representation of gene splicing PCR (Adapted from Gratchev http://www.methods.info/Methods/Mutagenesis/PCR_splicing.html). (TIF 90 kb)
Additional file 2: Table S1. Primers used for PCR mediated splicing, cloning and real-time gene expression of Lus10006353 and Lus10014148. (DOCX 16 kb)
Additional file 3: Figure S2. Circos map of 299 UGTs on the 15 flax chromosomes. Lines in the center of the map join duplicates. Scale for each chromosome is indicated in Mb. *UGT74S1*, *UGT74S4* (*LuS10014148*) and *UGT74S3* (*LuS10006353*) are marked with asterisks. (TIF 4003 kb)
Additional file 4: Table S2. List, location and coordinates of the 299 UGTs found in the flax genome. UGT locus corresponding to UGT74S1 is highlighted in bold red. (XLSX 25 kb)
Additional file 5: Figure S3. Distribution of UGT genes on the 15 chromosomes in the flax genome. The term “first and second copy” is arbitrary and was used only for graph presentation purpose. The fifteen chromosomes in the flax genome are indicated by Lu1 to Lu15. (TIF 40 kb)
Additional file 6: Figure S4. Phylogenetic relationship among 231 flax UGTs and depicting 15 groups. Sixty-eight UGTs with relatively short sequences were excluded. The four main clusters are indicated by alpha numeric numbers 1–4 and the different groups are indicated by alphabetic letters A-O around and inside the circular tree. *UGT74S1* belongs to a small gene family highlighted in red within group L and is marked with an asterisk. (TIF 8558 kb)
Additional file 7: Table S3.
*Ka*, *Ks, Ka/Ks* and divergence time for 15,616 UGT genes pairs after filtration. (XLSX 641 kb)
Additional file 8: Table S4. Pairwise matrix of flax UGT divergence time in MYA for 31 genes clustered in group “L” of the phylogenetic tree. The UGTs found in Lus10017825 and Lus10006351/Lus1006352 subclades are highlighted in green. The most recent and ancient divergence times are highlighted in blue and yellow, respectively. (XLSX 15 kb)
Additional file 9: Figure S5. Comparison of genomic DNA and full length coding regions for Lus10006353 (UGT74S3) and Lus10014148 (UGT74S4) as obtained by gene fusion PCR. A, Amplified exon I (678 bp) and exon II (738 bp) of Lus10006353 were fused to make the gene fusion product and compared to the genomic DNA amplicon; B, Amplified exon I (643 bp) and exon II (728 bp) of Lus10014148 were fused and similarly compared to its genomic DNA amplicon. Molecular ladder sizes are indicated by arrows. (TIF 83 kb)
Additional file 10: Figure S6. Sequence alignment between the fused spliced CDS from *Lus10006353 (UGT74S3)* and *Lus10014148 (UGT74S4)* and their respective corresponding genomic and transcript sequences reported in NCBI and Phytozome. A, Sequence alignment of the fused spliced *Lus10006353 (UGT74S3)* CDS with its amplified genomic region before splicing (genomic Lus10006353), its corresponding genomic sequences reported in NCBI and Phytozome (*JN088326.1* and Lus10006353.g, respectively), and its transcript sequence from Phytozome (Lus10006353). The genomic sequences show the 76 bp intron located between nucleotide positions 679 and 756 whereas the intron is missing in the published transcript sequence and in the fused spliced CDS reported in the current study; B, Sequence alignment of the fused spliced *Lus10014148 (UGT74S4)* CDS with its amplified region before splicing (genomic Lus10014148), its corresponding genomic sequences reported in NCBI and Phytozome (*JN088327.1* and Lus10014148.g, respectively), and its transcript sequence from Phytozome (Lus10014148). The genomic sequences show the 93 bp intron located between nucleotide position 643 and 737 whereas the intron is missing in the published transcript sequence and in the fused spliced CDS reported in the current study. (TIF 1168 kb)
Additional file 11: Figure S7. Protein sequence alignment between UGT74S1, Lus10006353 (UGT74S3) and Lus10014148 (UGT74S4). The PSPG region is indicated by a red box and where amino acid variations between the three proteins appear as a white indentation in the black box representing the consensus sequence. All amino acid positions were numbered according to their location in UGT74S1. (TIF 1731 kb)

## Abbreviations

CDS: Coding sequence
DAA: Day after anthesis
EMS: Ethyl methane sulfonate
*Ka*: Non-synonymous substitution
*Ks*: Synonymous substitution
MS: Mass spectrometry
MYA: Million year ago
NCBI: National center for biotechnology information
PSPG: Plant secondary product glycosyltransferase
SDG: Secoisolariciresinol diglucoside
SECO: Secoisolariciresinol
SMG: Secoisolariciresinol monoglucoside
SMGal: Secoisolariciresinol monogalactoside
TILLING: Targeted induced local lesion in genome
UDP: Uridine diphosphate
UGT: Uridine glycosyltransferases
UPLC: Ultra performance liquid chromatography
