# Supplementary figures and images for: UGT74S1 is the key player in controlling secoisolariciresinol diglucoside (SDG) formation in flax
Source: BMC Plant Biol. 2017 Feb 2;17:35. doi: 10.1186/s12870-017-0982-x (PMC5290659; doi:10.1186/s12870-017-0982-x)

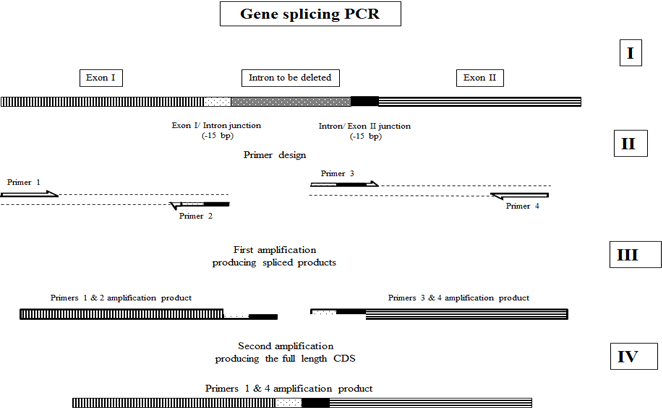

Supplement: Additional file 1: Figure S1. — Schematic representation of gene splicing PCR (Adapted from Gratchev http://www.methods.info/Methods/Mutagenesis/PCR_splicing.html). (TIF 90 kb) [file 12870_2017_982_MOESM1_ESM.tif]

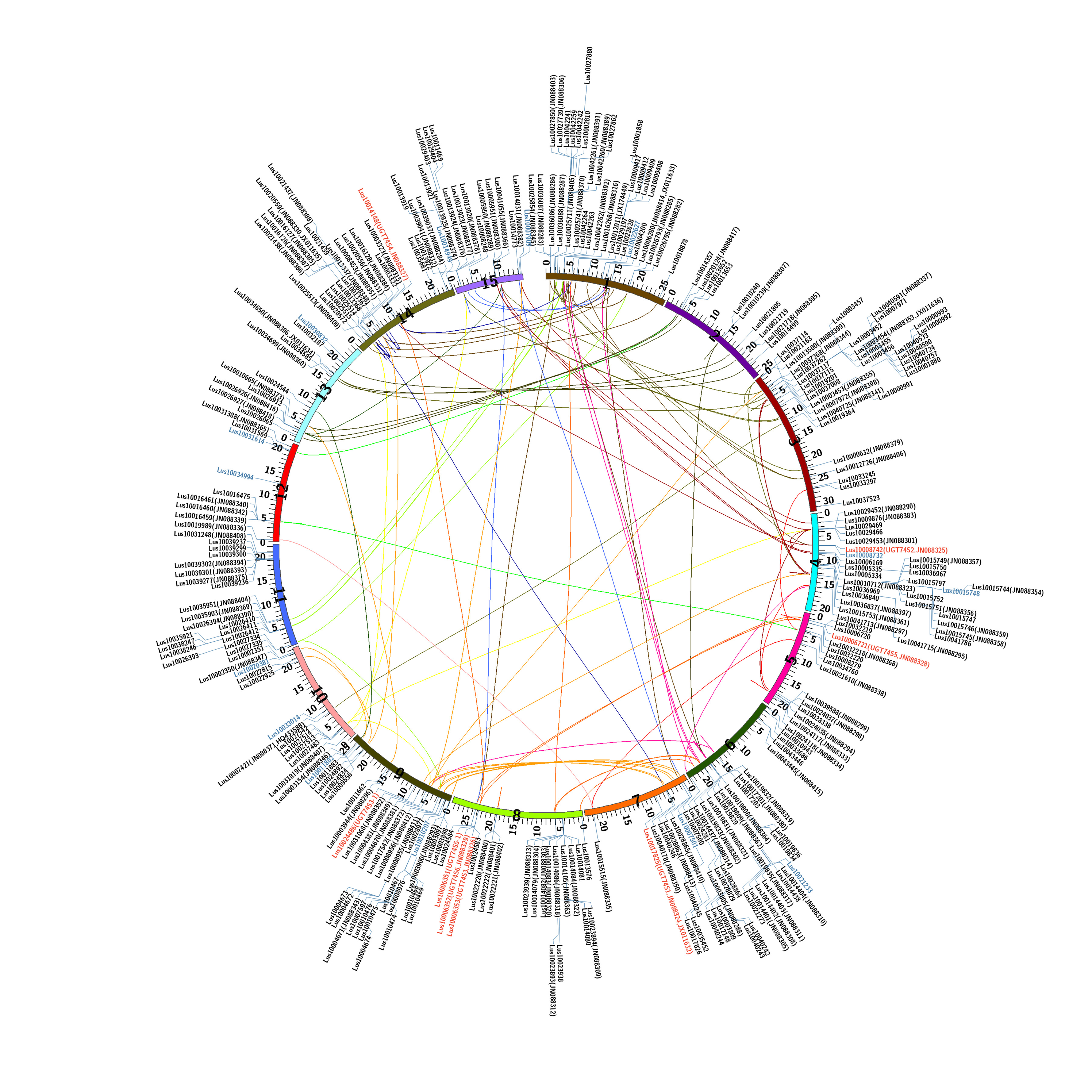

Supplement: Additional file 3: Figure S2. — Circos map of 299 UGTs on the 15 flax chromosomes. Lines in the center of the map join duplicates. Scale for each chromosome is indicated in Mb. UGT74S1, UGT74S4 (LuS10014148) and UGT74S3 (LuS10006353) are marked with asterisks. (TIF 4003 kb) [file 12870_2017_982_MOESM3_ESM.tif]

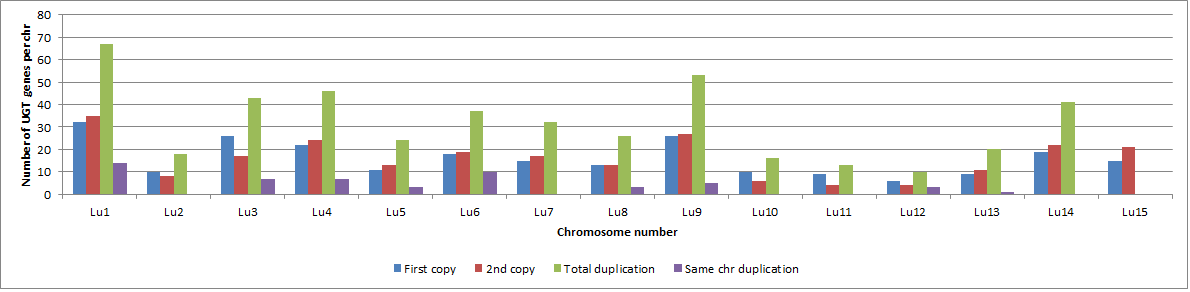

Supplement: Additional file 5: Figure S3. — Distribution of UGT genes on the 15 chromosomes in the flax genome. The term “first and second copy” is arbitrary and was used only for graph presentation purpose. The fifteen chromosomes in the flax genome are indicated by Lu1 to Lu15. (TIF 40 kb) [file 12870_2017_982_MOESM5_ESM.tif]

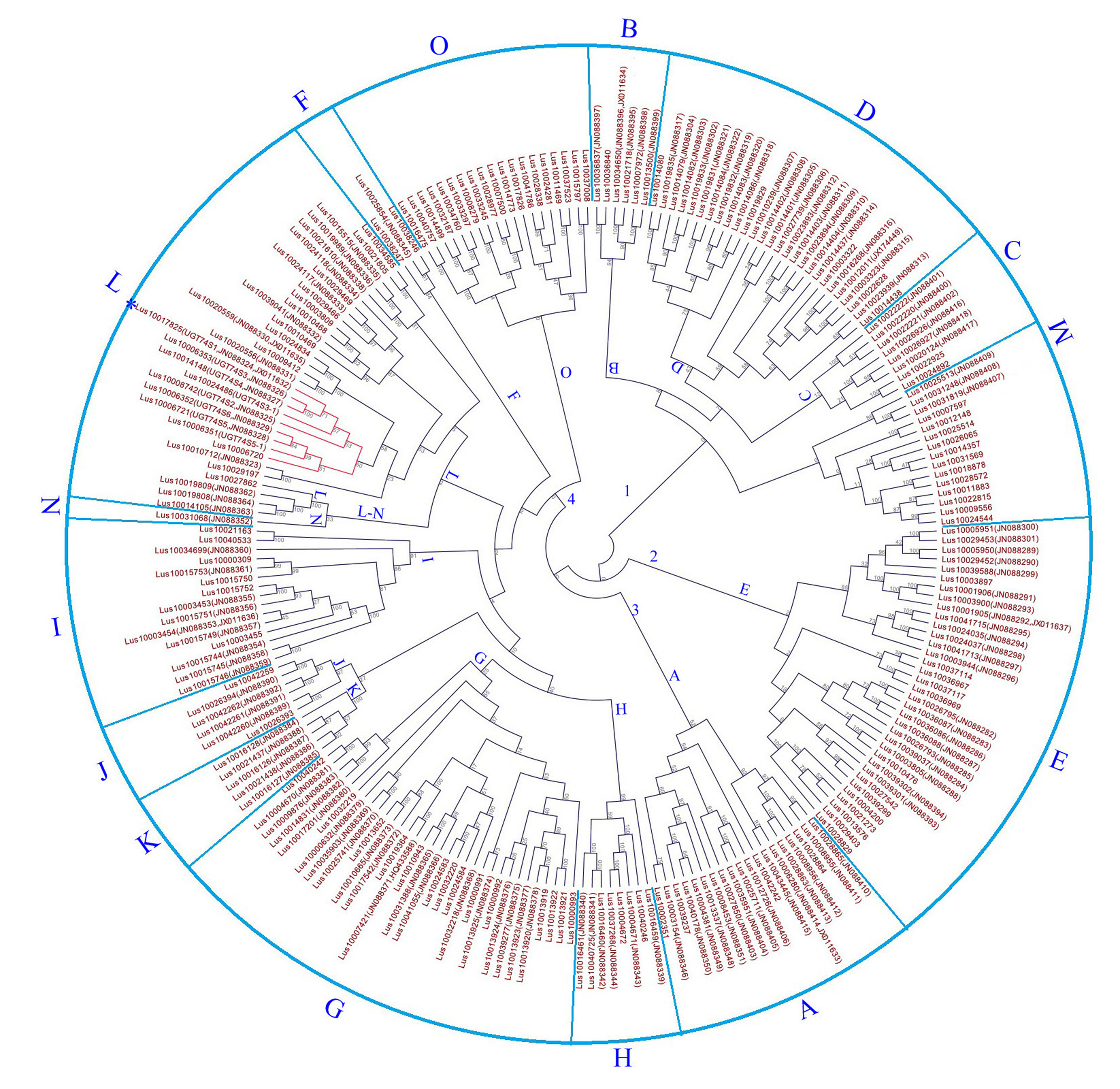

Supplement: Additional file 6: Figure S4. — Phylogenetic relationship among 231 flax UGTs and depicting 15 groups. Sixty-eight UGTs with relatively short sequences were excluded. The four main clusters are indicated by alpha numeric numbers 1–4 and the different groups are indicated by alphabetic letters A-O around and inside the circular tree. UGT74S1 belongs to a small gene family highlighted in red within group L and is marked with an asterisk. (TIF 8558 kb) [file 12870_2017_982_MOESM6_ESM.tif]

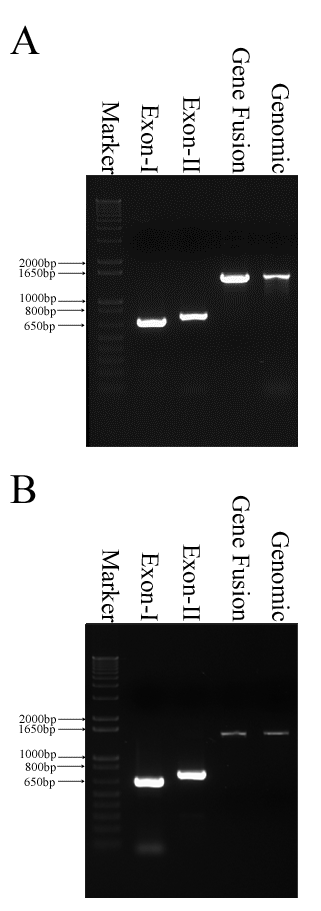

Supplement: Additional file 9: Figure S5. — Comparison of genomic DNA and full length coding regions for Lus10006353 (UGT74S3) and Lus10014148 (UGT74S4) as obtained by gene fusion PCR. A, Amplified exon I (678 bp) and exon II (738 bp) of Lus10006353 were fused to make the gene fusion product and compared to the genomic DNA amplicon; B, Amplified exon I (643 bp) and exon II (728 bp) of Lus10014148 were fused and similarly compared to its genomic DNA amplicon. Molecular ladder sizes are indicated by arrows. (TIF 83 kb) [file 12870_2017_982_MOESM9_ESM.tif]

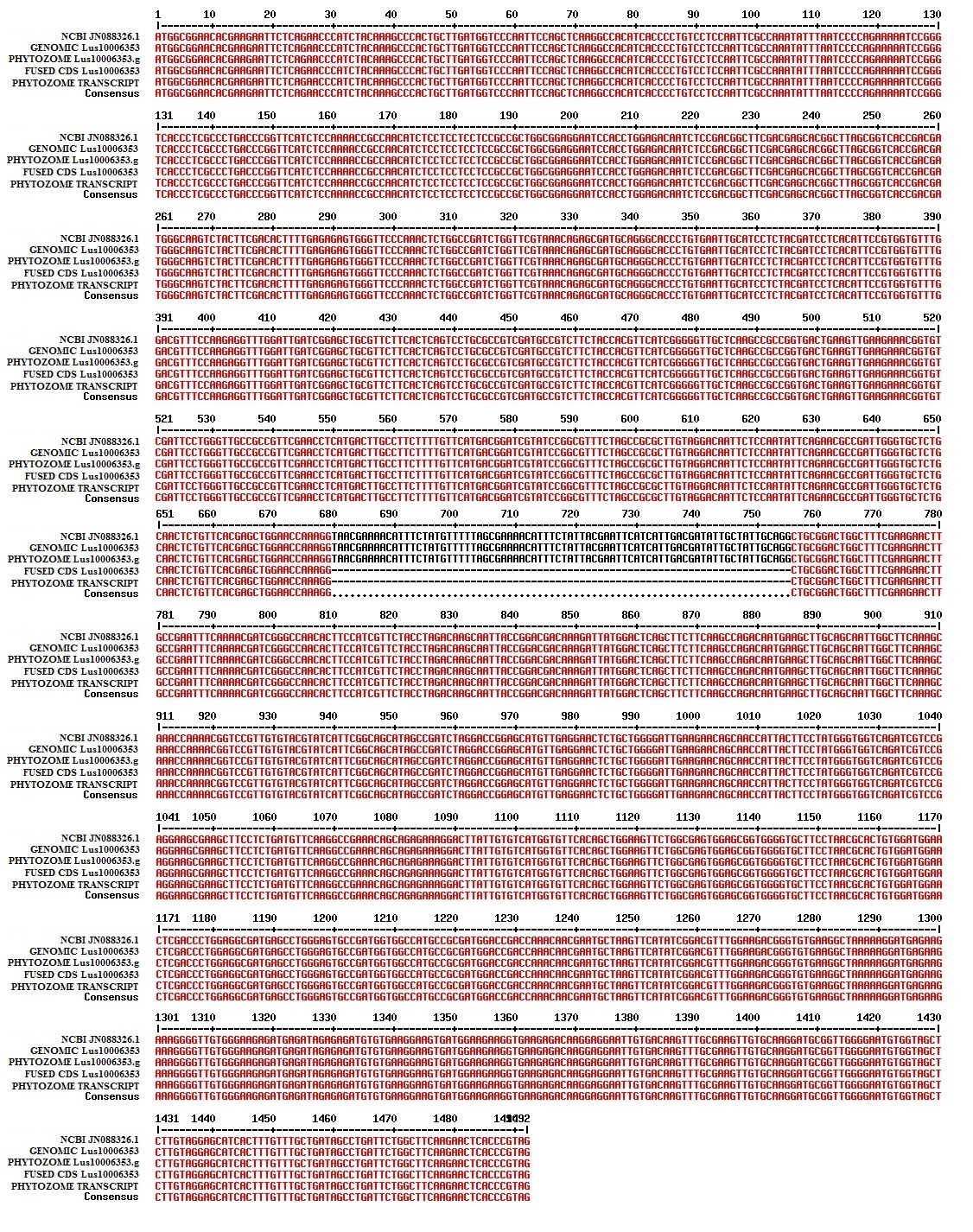

Supplement: Additional file 10: Figure S6. — Sequence alignment between the fused spliced CDS from Lus10006353 (UGT74S3) and Lus10014148 (UGT74S4) and their respective corresponding genomic and transcript sequences reported in NCBI and Phytozome. A, Sequence alignment of the fused spliced Lus10006353 (UGT74S3) CDS with its amplified genomic region before splicing (genomic Lus10006353), its corresponding genomic sequences reported in NCBI and Phytozome (JN088326.1 and Lus10006353.g, respectively), and its transcript sequence from Phytozome (Lus10006353). The genomic sequences show the 76 bp intron located between nucleotide positions 679 and 756 whereas the intron is missing in the published transcript sequence and in the fused spliced CDS reported in the current study; B, Sequence alignment of the fused spliced Lus10014148 (UGT74S4) CDS with its amplified region before splicing (genomic Lus10014148), its corresponding genomic sequences reported in NCBI and Phytozome (JN088327.1 and Lus10014148.g, respectively), and its transcript sequence from Phytozome (Lus10014148). The genomic sequences show the 93 bp intron located between nucleotide position 643 and 737 whereas the intron is missing in the published transcript sequence and in the fused spliced CDS reported in the current study. (TIF 1168 kb) [file 12870_2017_982_MOESM10_ESM.tif]

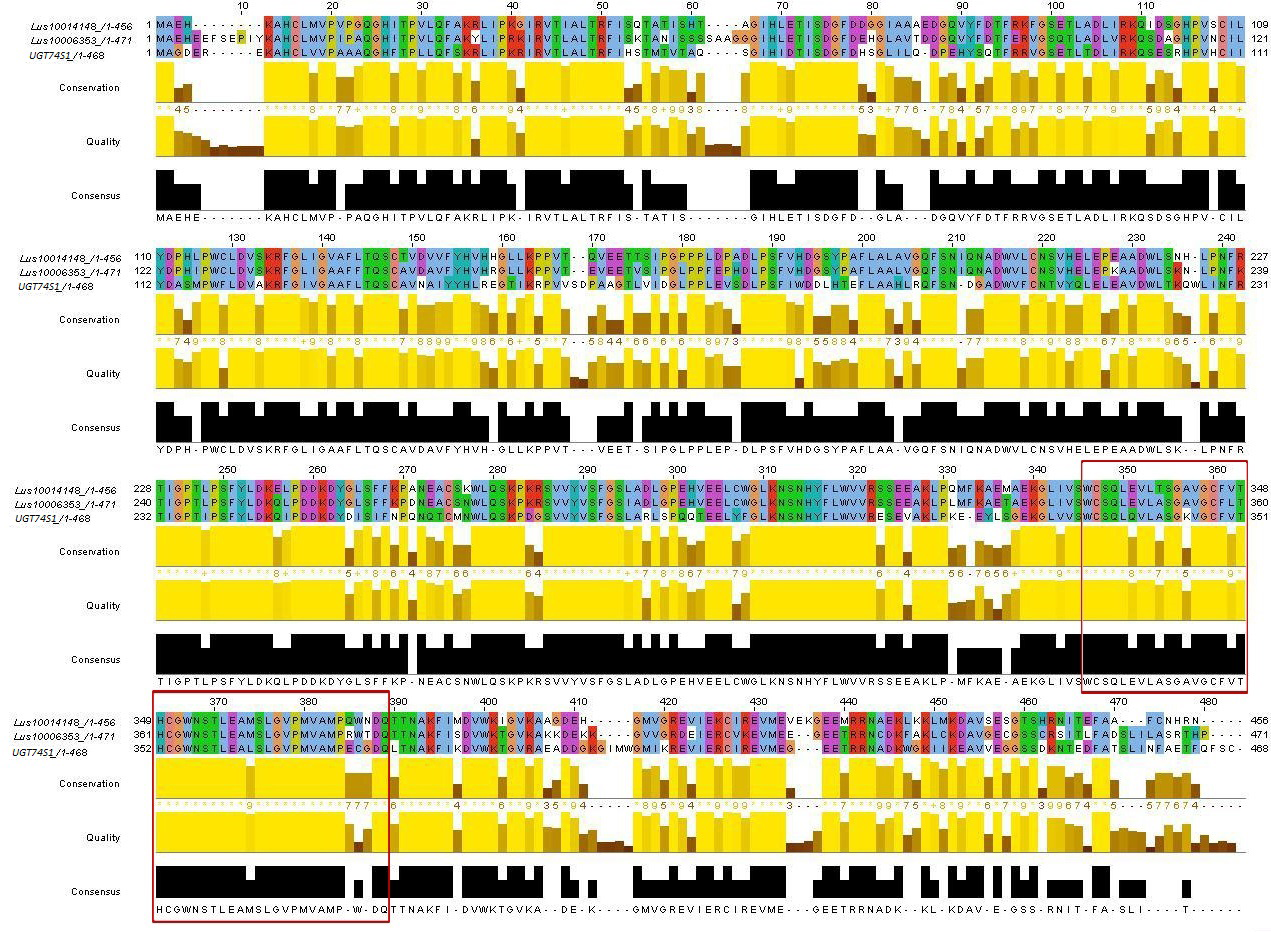

Supplement: Additional file 11: Figure S7. — Protein sequence alignment between UGT74S1, Lus10006353 (UGT74S3) and Lus10014148 (UGT74S4). The PSPG region is indicated by a red box and where amino acid variations between the three proteins appear as a white indentation in the black box representing the consensus sequence. All amino acid positions were numbered according to their location in UGT74S1. (TIF 1731 kb) [file 12870_2017_982_MOESM11_ESM.tif]
