# Supplementary material for: UGT74S1 is the key player in controlling secoisolariciresinol diglucoside (SDG) formation in flax
Source: BMC Plant Biol. 2017 Feb 2;17:35. doi: 10.1186/s12870-017-0982-x (PMC5290659; doi:10.1186/s12870-017-0982-x)
Supplement: Additional file 2: Table S1. — Primers used for PCR mediated splicing, cloning and real-time gene expression of Lus10006353 and Lus10014148. (DOCX 16 kb) [file 12870_2017_982_MOESM2_ESM.docx]

**Additional File 2: Table S1 -** Primers used for PCR mediated splicing, cloning and real-time gene expression of Lus10006353 and Lus10014148

| Application | Primer name | Primer sequence (5’-3’) | |
| --- | --- | --- | --- |
|  |  | Forward | Reverse |
| Splicing | Lus10006353 F/R | ATGGCGGAACACGAAGAATTCTCAGAAC | CTACGGGTGAGTTCTTGAAGCCAG |
|  | Lus10014148 F/R | ATGGCGGAACACAAAGCCCACTGC | TTAGTTTCTATGATTACAGAATGCAGC |
|  | Lus10006353 exon II-F/ exon I-R | TCGACCTTGGTTTCCCTGCGGACTGGCTTT | AAAGCCAGTCCGCAGCCTTTGGTTCCAGCT |
|  | Lus10004148 exon II-F/ exon I-R | CTCGACCTTGGCCTCCCGCGGACTGGCTCT | AGAGCCAGTCCGCGGCTCCGGTTCCAGCTC |
| Cloning | Lus10006353 BamH1-F/ XhoI-R | AAAGGATCCATGGCGGAACACGAAG | TTTCTCGAGCTACGGGTGAGTTCTTG |
|  | Lus10014148 NotI-F/ XhoI-R | AAAGCGGCCGCATGGCGGAACACAAAGC | TTTCTCGAGTTAGTTTCTATGATTAC |
| Real-time | Lus10006353 RT-F/ Lus10006353 RT-R | GACGGGTGTGAAGGCTAAAA | GAGCTACCACATTCCCCAAC |
|  | Lus10014148 RT-F/ Lus10014148 RT-R | GGACTGATCGTGTCATGGTG | GCAGCCTTCACACCAATCTT |
|  | UGT74S1 F/UGT74S1 R | GGATGGAACTCGACTCTGGA | CTTTCCCCACTTGTCAGCAT |
|  | rRNA F/ rRNA R | ATTCGGCCCGTCTTGAAACA | GGGCCTCCACCAGAGTTTCC |

F, Forward, R, reverse, RT, Real-time
